# Plasma membrane proteomes of differentially matured dendritic cells identified by LC–MS/MS combined with iTRAQ labelling

**DOI:** 10.1016/j.jprot.2011.10.010

**Published:** 2012-01-04

**Authors:** Stéphanie Ferret-Bernard, William Castro-Borges, Adam A. Dowle, David E. Sanin, Peter C. Cook, Joseph D. Turner, Andrew S. MacDonald, Jerry R. Thomas, Adrian P. Mountford

**Affiliations:** a Department of Biology, University of York, York, YO10 5DD, UK; b Centre of Excellence in Mass Spectrometry, University of York, York, YO10 5DD, UK; c Institute of Immunology and Infection Research, School of Biological Sciences, University of Edinburgh, UK

**Keywords:** 0–3hRP, zero-to-three hours released proteins, Arp2/3, actin-related protein 2/3 complex, BM, bone marrow, CD, cluster of differentiation, DC, dendritic cell, E/S, excretory/secretory, GAPDH, glyceraldehyde 3-phosphate dehydrogenase, GM-CSF, granulocyte–macrophage colony-stimulating factor, GNBP, guanine nucleotide-binding protein, LPS, lipopolysaccharide, MFI, median fluorescence intensity, NAP-22, 22 kDa neuronal tissue-enriched acidic protein, PAMP, pathogen-associated molecular pattern, PRR, pattern recognition receptor, SEA, schistosome egg antigen, Th, T helper, Dendritic cell, iTRAQ, Parasitic helminth, Plasma membrane proteomics

## Abstract

Dendritic cells (DCs) play a pivotal role in polarising Th lymphocyte subsets but it is unclear what molecular events occur when DCs generate Th2-type responses. Here, we analysed plasma membrane-enriched fractions from immature, pro-Th1 and pro-Th2 DCs and used a combination of iTRAQ labelling and LC–MS/MS to quantify changes in the proteomes. Analysis was performed on triplicate biological samples and changes verified by flow cytometry. MHC class II molecules and CD29 were up-regulated in pro-Th1 DCs whilst CD18 and CD44 were up-regulated in pro-Th2 DCs. One of the most down-regulated molecules in pro-Th1 DCs was YM-1 whilst the greatest decrease in pro-Th2 DCs was NAP-22. Other molecules up-regulated in pro-Th2 DC compared to pro-Th1 DCs included some potentially involved in protein folding during antigen processing (clathrin and Rab-7), whilst other non-membrane proteins such as enzymes/transporters related to cell metabolism (malate dehydrogenase, pyruvate kinase, and ATPase Na^+^/K^+^) were also recorded. This suggests that pro-Th2 DCs are more metabolically active while pro-Th1 DCs have a mature ‘end state’. Overall, although several molecules were preferentially expressed on pro-Th2 DCs, our proteomics data support the view of a ‘limited maturation’ of pro-Th2 DCs compared to pro-Th1 DCs.

## Graphical abstract

**Figure:**
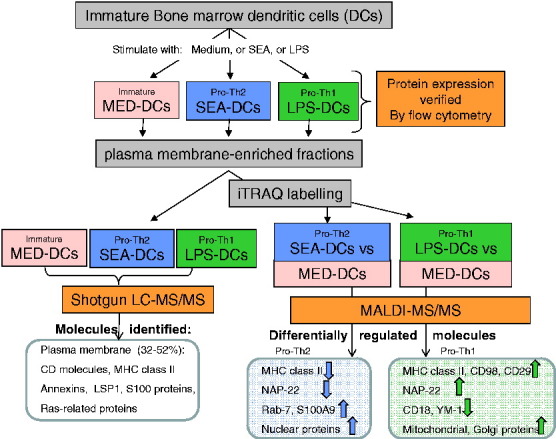

**Highlights:**

► Analysis of plasma membrane-enriched proteomes of differentially matured DCs. ► Relative expression determined by iTRAQ labelling combined with LC–MS/MS. ► Differential expression of immune associated proteins by pro-Th2 *versus* pro-Th1 DCs. ► Data support ‘limited maturation’ but not ‘unique’ phenotype of pro-Th2 DCs.

## 1 Introduction

Dendritic cells (DCs) are critical in development of immunity against pathogens [1] and are able to interpret different pathogen-inherent signals to play a pivotal role in polarising Th lymphocyte subsets [2]. In general, pathogen-associated molecular patterns (PAMPs) that drive DCs to promote Th1-type responses, such as bacterial lipopolysaccharide (LPS), bind to pattern recognition receptors (PRRs) on the DC plasma membrane leading to subsequent activation and maturation of the DCs. Conversely, molecules from helminths, which are potent inducers of Th2-type responses, stimulate DCs with a ‘modified’ phenotype [3–7] but it is unclear how/why these DCs promote Th2-type immunity. Pro-Th2 DCs may have a distinct phenotype, defined by a unique profile of signature molecules [4,5], or may resemble immature DCs, stimulating Th2 polarisation *via* a ‘default’ pathway in the absence of Th1-inducing stimuli [8]. An ‘inhibition model’ in which Th2-stimuli inhibit Th1 polarisation by DCs through competitive signalling pathways has also been proposed [9].

Changes in DC gene expression correlate poorly with changes in the level of protein expression [10,11]. Therefore, as protein expression is a better indicator of cell phenotype and function, a number of studies have examined the proteomes of differentially matured DCs [12–15]. For example, 2-DE and MS/MS have revealed changes in the proteome of pro-Th1 DCs matured with LPS *versus* pro-Th2 DCs stimulated with excretory/secretory (E/S) material from a parasitic helminth *Schistosoma mansoni* [14]. This E/S material, released by the parasite during infection and known as ‘0**–**3hRP’, is an important stimulant of innate immune cells in the skin [16,17] enabling DCs to promote Th2 responses *in vitro* and *in vivo* [7]. Proteomic analysis of pro-Th1 DCs revealed up-regulated expression of cytoskeletal proteins and chaperone molecules whereas pro-Th2 DCs, stimulated with 0**–**3hRP, exhibited a proteome intermediate between that of immature DCs and pro-Th1 DCs; thus termed a ‘limited maturation’ phenotype [14]. As soluble cytosolic molecules dominated the cell extracts used in the study, immune-associated proteins from the plasma membrane (*e.g.* PRRs, adhesion molecules, MHC complexes and costimulatory molecules) were not readily detected. Although such molecules are likely to be highly relevant with respect to differential maturation of DCs, their low abundance and hydrophobic nature makes them difficult to isolate for proteomic characterisation.

In order to ‘home in’ on the detection of specific plasma membrane proteins which are likely to be important in differential DC maturation, we compared proteins enriched from plasma membranes of immature DCs, pro-Th1 DCs stimulated with LPS and pro-Th2 DCs stimulated with schistosome egg antigen (SEA) which is a well characterised pro-Th2 helminth product [3]. First, DC plasma membrane-enriched fractions were analysed by shotgun LC–MS/MS to establish a list of proteins associated with DCs. Second, a gel-free technique using iTRAQ [18,19] was used to quantify changes in protein expression following differential DC maturation. By performing three biological replicates of each type of DCs and confirming proteomic data by flow cytometry, we identified a number of proteins that were differentially expressed by pro-Th1 *versus* pro-Th2 DCs.

## 2 Materials and methods

### 2.1 Generation and maturation of DCs from bone marrow

Bone marrow-derived dendritic cells (BM-DCs) from female C57BL/6 strain mice were cultured in RPMI medium containing 10% low endotoxin FCS plus 20 ng/mL GM-CSF (Peprotech, London, UK) as previously described [7,14]. All experimental procedures were undertaken with the guidelines of the United Kingdom Animal's Scientific Procedures Act 1986 and approved by the University of York Ethics committee. On day 6, immature BM-DCs were seeded at 1 *×* 10^6^/mL and cultured for 18 h alone (MED-DCs), or in the presence of 40 μg/mL SEA [3] (SEA-DCs), or 10 ng/mL LPS (from *Escherichia coli* strain 0111:B4, Sigma-Aldrich, Poole, UK; LPS-DCs) [14]. After overnight culture, cells were harvested and prepared for proteomic analysis.

### 2.2 Preparation of DC plasma membrane-enriched fractions

Plasma membrane proteins from MED-DCs, SEA-DCs and LPS-DCs were extracted and purified using a plasma membrane protein extraction kit (BioVision, Mountain View, USA). All steps were performed at 4 °C. Briefly, BM-DCs were mechanically homogenised in an ice-cold glass cell grinder and then spun at 700 *g*. The resulting supernatants were spun at 10,000 *g* for 30 min to yield total membrane protein (*i.e.* plasma and cellular organelle membranes) enriched pellets which were re-suspended in ‘Upper Phase solution’ and mixed with an equal volume of ‘Lower Phase solution’ before centrifugation at 1000 × *g* for 5 min. The upper phase was spun at 25,000 *g* for 10 min, and the resulting plasma membrane-enriched pellet solubilised in 0.5% Triton X-100. Total protein content was assessed by densitometry of SYPRO Ruby stained 1-D electrophoresis gels (NuPAGE 4–12%) against known quantities of cytosolic fractions as standards separated on the same gel.

### 2.3 Digestion and iTRAQ labelling

Plasma membrane-enriched fractions (35–50 μg) were reduced with 2 mM tris-(2-carboxyethyl)phosphine in 0.5 M triethylammonium bicarbonate (Sigma-Aldrich) pH8.5, at 60 °C for 1 h, alkylated with 10 mM methyl methanethiosulfonate (Sigma-Aldrich) at room temperature for 10 min and digested overnight with sequencing-grade porcine trypsin (Promega, Madison, USA) at 37 °C. The iTRAQ labelling reagents (114, 115, and 116; Applied Biosystems, Framingham, USA) were reconstituted in isopropanol and added to the digests. After 2 h, labelled peptides were combined and purified using cation-exchange and C18 cartridges. Although iTRAQ allows for the multiplexing of several samples in a single run, comparison is performed in a pair-wise manner. In this respect, a common reference sample between iTRAQ analyses is essential. However, a pooled standard is not universally required if the samples are suitably similar, as in our study, where MED-DC is taken as a common standard across all runs [20].

### 2.4 LC–MS/MS

Peptides were separated using a Dionex polystyrene-divinylbenzene column and fractions collected directly onto a target plate with addition of CHCA matrix. Positive-ion MALDI mass spectra were acquired using an Applied Biosystems 4700 Proteomics Analyzer in reflectron mode over a mass range of *m/z* 800–4000. Monoisotopic masses were obtained from centroids of raw, unsmoothed data. The 20 most intense peaks with a S/N ≥ 50 from each fraction were selected for CID-MS/MS using collision energy of 1 keV, air as collision gas, precursor mass window set to a relative resolution of 50 and metastable suppressor enabled. MS/MS spectra were baseline-subtracted (peak width 50) and smoothed (Savitsky–Golay; three points; polynomial order 4); peak detection used S/N ≥ 5, local noise window 50 *m/z* and minimum peak width 2.9 bins. Mascot peak list files were generated using the TS2Mascot utility (Matrix Science, version 1.0.0) with S/N ≥ 10.

### 2.5 Protein identification and quantification

Peak lists were searched against the CDS (Celera Discovery System™, KBMS3.0.20040121) mouse database (65,307 sequences; 23,201,165 residues) using Mascot (Matrix Science Ltd., version 2.1). Search criteria specified: Enzyme, Trypsin; Maximum missed cleavages, 1; Fixed modifications, Methylthio (C); Variable modifications, Oxidation (M); Peptide tolerance, 0.3 Da; MS/MS tolerance, 0.3 Da; Instrument, MALDI-TOF-TOF. When searching iTRAQ peak lists the fixed modifications, iTRAQ4plex (N-term) and iTRAQ4plex (K), and the variable modification iTRAQ4plex (Y), were also specified. The Mascot significance threshold for protein identification was adjusted in each search to give a false discovery rate of approximately 1%. Only peptides that met or exceeded their identity score at this significance threshold and had an expect score less than 0.05 were accepted, including single-peptide protein matches. Where peptides match to multiple members of a protein family, the protein that tops the Mascot protein group and hence has the highest number of unique peptides, is reported. Mascot was used for iTRAQ quantification with these options: normalisation by median ratio; automatic outlier removal; median ratios for protein ratios. Fold changes were accepted only with three peptides. Each iTRAQ experiment included three biological replicates.

### 2.6 Sub-cellular and Gene Ontology classification of proteins

Sub-cellular classifications were performed with Gene Ontology classification (http://www.ebi.ac.uk/GOA/) according to the accession number of protein identities in Uniprot. If no classification was found, Mouse Genome Informatics website (http://www.informatics.jax.org/) and the Proteome Analyst v3.0 (http://pa.cs.ualberta.ca:8080/pa/) were used. Finally, if no prediction was available, we referred to published literature. Proteins identified as ‘plasma membrane’ included formations such as lipid rafts, endosomes, phagocytic cups and phagosomes. ‘Cytosol’ comprised cytosolic, cytoplasmic proteins and vesicles. Cytoskeletal proteins were given a particular column although most are also cytosolic. Ribosome proteins were classified as belonging to the ‘ER’, whilst proteins associated with the Golgi apparatus, late endosomes and lysosomes, were defined as ‘Golgi’. However, classification of proteins into discrete compartments is arbitrary depending upon original allocation by bio-informatic interrogation and may be best allocated to more than one classification. Protein hits that were observed to be differentially regulated (*i.e.* SEA-DCs *vs* MED-DCs, and LPS-DCs *vs* MED-DCs) were analysed according to Gene Ontology (GO) classification using Visual Annotation Display (VLAD; proto.informatics.jax.org/prototypes/vlad-1.0.3/). Significant hits were selected on the basis of their p-value and the number of proteins(k) found within each category.

### 2.7 Validation of protein expression by flow cytometry

Differentially matured DCs were blocked with anti-CD16/32 mAb (BD Pharmingen, Oxford, UK) in PBS (supplemented with 1% FCS and 5 mM EDTA) and subsequently labelled with the following conjugated mAbs against: CD18-biotin (M18/2), CD29-PE (HMb1-1), CD44-FITC (1C10), CD98-FITC (RL388), IA/IE-FITC (M5/114.15.2), Galectin-3-biotin (M3/38; all from eBioscience, Hatfield, UK), I-A^b^ biotin (28-16-8S; Caltag Medsystems, Buckingham, UK) and CD301b-AF647 (ER-MP23; AbD Serotec, Kidlington, UK). Biotin conjugated antibodies were probed with streptavidin-APC (Caltag Medsystems). Unlabelled rabbit polyclonal antibodies were used to probe for S100A10 (Abcam, Cambridge, UK), YM-1 (STEMCELL Technologies, Grenoble, France) and Rab-7 (Sigma-Aldrich), and were subsequently detected with anti-rabbit AF488 antibody (eBioscience). All antibody concentrations were optimised and labelling was performed alongside relevant isotype controls. Flow cytometric acquisition and analysis was performed using a Cyan ADP analyser with Summit v4.3 (DakoCytomation, UK). Data were plotted as means of the median fluorescence intensity (MFI) of three separate DC cultures for each maturation stimulus.

## 3 Results

### 3.1 Sub-cellular classification of proteins identified by shotgun LC–MS/MS

**Fig. 1:**
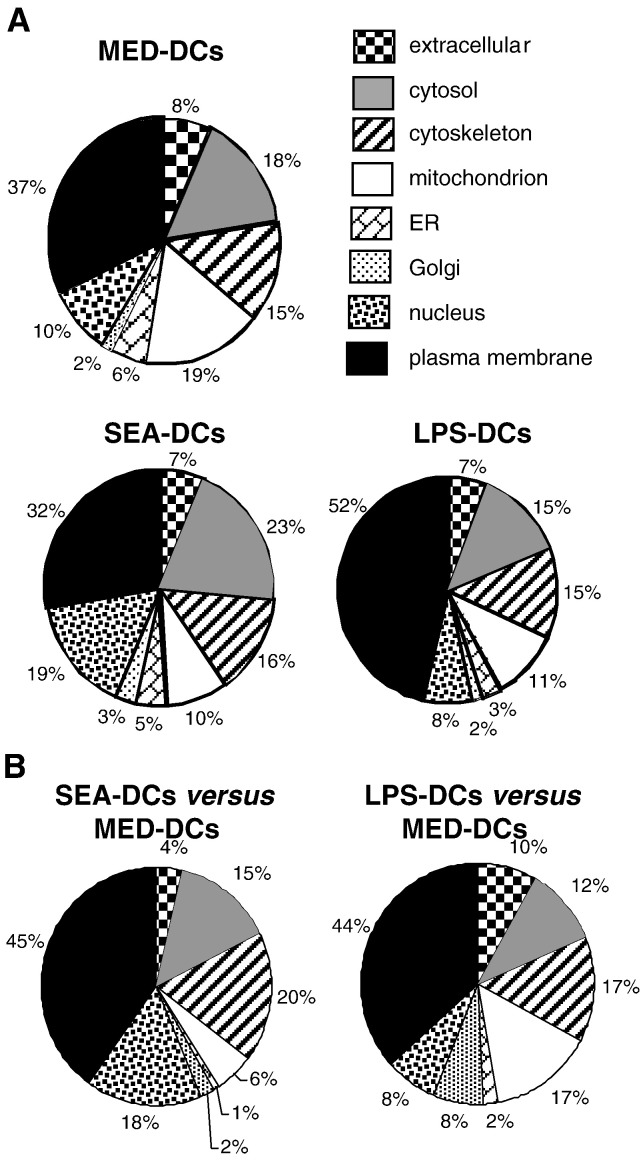
Sub-cellular classification of the total identified proteins. Pie-charts showing the classification of total protein identities in plasma membrane fractions obtained from MED-DCs, SEA-DCs and LPS-DCs determined by LC–MS/MS (A). Pie-charts showing the classification of the total hits obtained from three technical replicates of iTRAQ experiments comparing SEA-DCs or LPS-DCs *versus* MED-DCs (B).

Initial shotgun LC–MS/MS proteomic analyses of DC fractions were performed to validate the isolation technique. In MED-DCs, 119 significant hits were identified (Supp. Table 1) of which 37% were classified by Gene Ontology as plasma membrane (Fig. 1A). In SEA-DCs and LPS-DCs, 149 and 61 proteins respectively were identified (Supp. Tables 2 and 3) with 32% and 52% from the plasma membrane (Fig. 1A). The fractions also contained cytosolic (19%), cytoskeletal (15%) and nuclear proteins whilst extracellular, ER and Golgi proteins were only minor components. In six different purification experiments, the enrichment for plasma membrane proteins in the fractions, compared to the total cell, was 23.4 ± 3.1. Consequently, we concluded that the isolation technique yielded fractions greatly enriched in plasma membrane molecules.

Plasma membrane molecules (shaded rows in bold text of Supp. Tables 1, 2, and 3) comprised those with a CD prefix (18%) and ras-related proteins (rho, rac, rab and rap; 17%) which were highly represented from all three types of DC. Other abundant molecules were MHC class I and II molecules for LPS-DCs (13%) and guanine nucleotide-binding proteins (GNBPs) in MED-DCs (11%). Annexins (1, 2, 4 and 5) and S100 proteins represented 10% and 5% respectively, of plasma membrane proteomes of all DC types.

### 3.2 Plasma membrane proteomes of differentially matured DCs after iTRAQ labelling

The number of hits identified after iTRAQ labelling in SEA-DCs *versus* MED-DCs, and LPS-DCs *versus* MED-DCs increased compared to the number of hits in SEA-DCs and LPS-DCs identified solely by LC–MS/MS (+ 66% and + 46% respectively; Supp. Tables 4, 5 *versus* 1, 2, 3). This resulted in expanded DC proteome coverage and database searching of multiple peptides per protein improved the confidence of the identifications. Nevertheless, the proportion of hits identified after iTRAQ labelling and classed as plasma membrane were similar to that revealed by shotgun LC–MS/MS.

Compilation of hits identified in the three separate iTRAQ experiments of SEA-DCs *versus* MED-DCs showed that 222 proteins were significantly identified with 45% being classed as plasma membrane components (Fig. 1B, Supp. Table 4) while a similar proportion (44%) was identified in LPS-DCs *versus* MED-DCs (Fig. 1B, Supp. Table 5). Overall, a greater proportion of hits were identified as nuclear proteins in SEA-DCs *versus* MED-DCs (18%) compared to LPS-DCs *versus* MED-DCs (8%) whereas a greater proportion of proteins in LPS-DCs *versus* MED-DCs were mitochondrial, extracellular and associated with the Golgi apparatus.

### 3.3 Up- or down-regulated proteins from differentially matured DC membrane-enriched proteomes

**Fig. 2:**
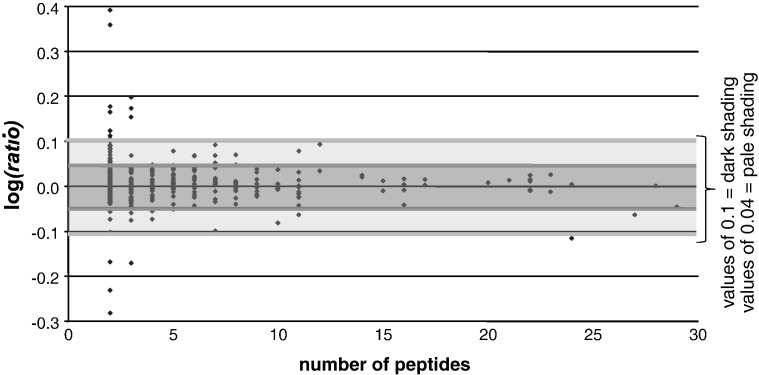
Whetton's plot. iTRAQ reporter-ion *ratios* for all proteins in the control *versus* control comparisons using the 114 and 116 reagents. A total of 355 protein *ratios* were plotted against the number of peptides that contributed to the *ratio*. Shading zones represent two levels of threshold: at log*(ratio)* values of ± 0.1 (*ratios* 0.79–1.26 in mid-grey) and a less stringent threshold of ± 0.04 (*ratios* 0.9–1.1 in light grey).

In order to assess variance in iTRAQ quantification experiments, Spooncer et al. [21] devised a form of analysis to compare separate preparations of the same biological material [22]. *Ratios* obtained by comparing separate preparations of the same material plotted against the number of peptides identified (Fig. 2) should cluster around unity (*i.e.* protein levels should be the same between replicates). The extent to which values deviate from unity is an indication of the biological and technical variance. This approach provided a powerful visual indication of where to set the significance threshold.

**Fig. 3:**
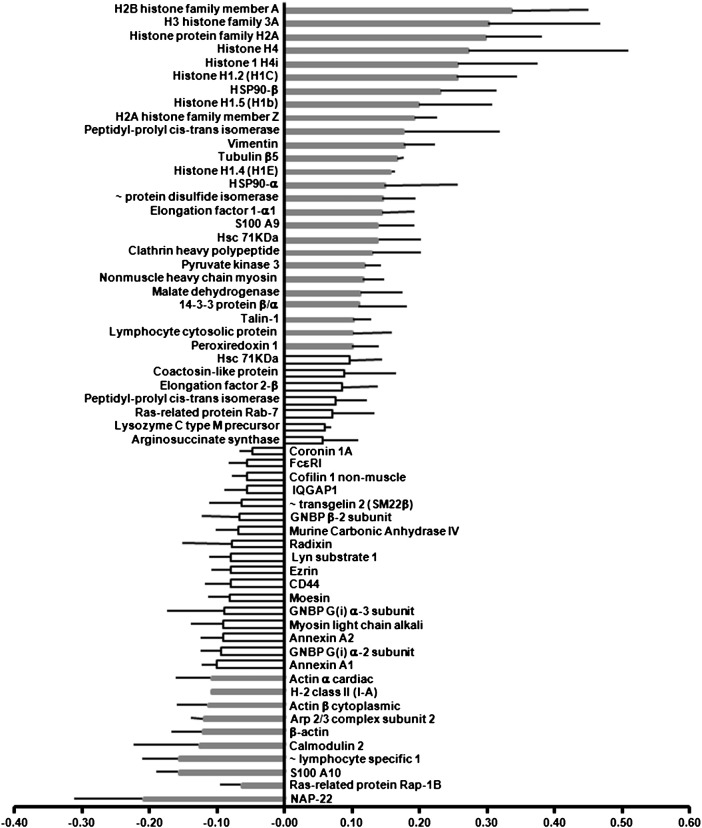
Changes on plasma membrane enriched-proteome of SEA-DCs *versus* MED-DCs. Up- and down-regulation of DC proteins as average log(*ratio*) ± standard error-95%, obtained from three iTRAQ experiments with criteria for acceptance clearly stated in the Results section.

**Fig. 4:**
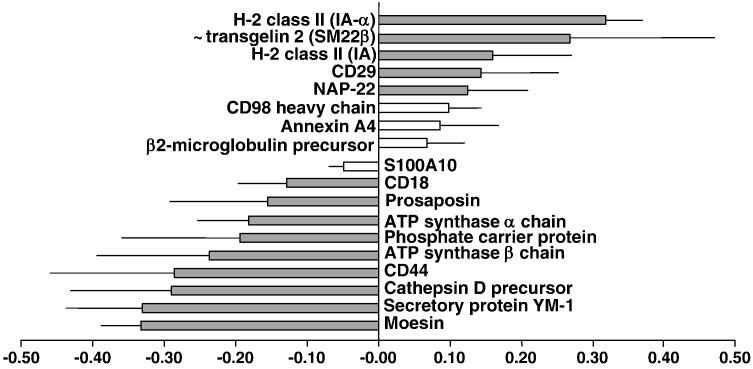
Changes on plasma membrane enriched-proteome of LPS-DCs *versus* MED-DCs. Up- and down-regulation of DC proteins as average log (*ratio*) ± standard error-95%, obtained from three iTRAQ experiments with criteria for acceptance clearly stated in the Results section.

Our iTRAQ experimental design consisted of six labelling experiments (Table 1), in which three preparations of SEA-DCs and three preparations of LPS-DCs were analysed. MED-DC fractions were labelled with 114 and 116 iTRAQ reagents to provide technical replicates whilst SEA-DC or LPS-DC preparations were labelled with 115. All the protein *ratios* from the six control–control (114:116) comparisons (355 in total) are shown in Fig. 2. As a more stringent threshold, we chose log*(ratio)* values of ± 0.1, which correspond to *ratios* of 1.26 and 0.79; a less stringent threshold of ± 0.04, which correspond to *ratios* of 1.1 and 0.9 was also used. Only changes where the average log(*ratio*) ± SEM-95% exceeded these thresholds were considered significant as given for SEA-DCs *versus* MED-DCs (Fig. 3, Supp. Table 6) and LPS-DCs *versus* MED-DCs (Fig. 4, Supp. Table 7). The more stringent threshold is shown as shaded bars and rows.

In SEA-DCs *versus* MED-DCs, the greatest up-regulation was for nuclear proteins (mainly histones) while several proteins associated with antigen uptake, processing and presentation (chaperones, clathrin, enzymes and the small GTPase Rab-7) were also up-regulated (Fig. 3, Supp. Table 6). Other molecules which were significantly up-regulated included: S100A9, 14-3-3β/α (part of TGFβ family), lymphocyte cytosolic protein 1 and lysozyme C. In contrast, decreased expression was observed for FcεRI, Rap-1B, hematopoietic cell specific lyn substrate 1, CD44, several annexins, MHC class II molecule (I-A), subunit 2 of actin-related protein 2/3 (Arp2/3) complex, lymphocyte-specific 1 protein (LSP1) and S100A10 (Fig. 3, Supp. Table 6). Down-regulation was also recorded for certain cytoskeletal proteins (ezrin, moesin, myosin and several isoforms of actin). The most pronounced down-regulation affected a protein not expected to be expressed by DCs: NAP-22 (22 kDa neuronal tissue-enriched acidic protein). Finally, several immune-related proteins were expressed at similar levels between SEA-DCs and MED-DCs (Fig. 3) including the integrins CD11b and CD18, the macrophage lectin CD301b, CD98 heavy chain, CD45, as well as MHC class I molecules (H-2D^b^ and H-2K^b^) and their associated β2-microglobulin. Also expressed at similar levels was the subunit 4 of Arp2/3 complex, proteins involved in general cell metabolism and the ras-related proteins Rac-1, -2, -3 together with cdc42.

Several proteins were highly up-regulated in LPS-DCs *versus* MED-DCs (Fig. 4, Supp. Table 7) including MHC class II molecules (IA-α representing the highest increase), CD98 heavy chain, β2-microglobulin and transgelin 2. Intriguingly, NAP-22 was up-regulated in LPS-DCs *versus* MED-DCs (it was down-regulated in SEA-DCs *versus* MED-DCs). The integrin CD29 (β subunit of VLA-4) was also up-regulated but not identified between SEA-DCs *versus* MED-DCs. Down-regulation affected S100A10, moesin and CD44, as also reported for SEA-DCs *versus* MED-DCs. Additionally, we observed specific decreased expression of CD18, both chains of ATP synthase and the precursor of cathepsin D. Significantly, expression of the secretory protein YM-1, associated with ‘alternatively-activated’ macrophages, was also highly down-regulated in LPS-DCs *versus* MED-DCs. Proteins whose expression was unchanged between LPS-DCs *versus* MED-DCs were GAPDH, several ras-related proteins (including Rab-7), several actin isoforms and galectin-3.

Analysis of protein classification according to GO terms revealed that SEA-DCs had numerous (k = 17) up-regulated proteins related to cellular metabolism, in particular Nucleoside phosphate metabolic processing (Table 2). These cells also exhibited up-regulated proteins associated with cytoskeleton remodelling specifically a number linked to the formation of cell projections (k = 13). On the other hand, SEA-DCs had down-regulated proteins associated with responses to chemical stimulation (k = 10) and GTPase activity (k = 5). Interestingly, down-regulated protein hits also mapped to those associated with antigen processing, and regulation of the adaptive immune response. In contrast, up-regulated proteins in LPS-DCs mapped clearly to antigen presentation and the MHC protein complex, whilst down-regulated proteins were associated with transmembrane transporter activity, catabolic processes and responses to wound healing (all k = 3).

### 3.4 Extrapolated changes between pro-Th2 DCs and pro-Th1 DCs

As we could not directly compare SEA-DCs *versus* LPS-DCs (due to the constraints of the availability of only 3 iTRAQ labels), we made an extrapolation of the above data in order to compare the protein expression changes between pro-Th2 and pro-Th1 DCs (Table 3). Amongst the proteins identified both in SEA-DCs *versus* MED-DCs and LPS-DCs *versus* MED-DCs, moesin, CD18, CD44 and Rab-7 were increased in SEA-DCs compared to LPS-DCs. Conversely, transgelin 2, NAP-22, MHC class II, annexin A1 and A4, β2-microglobulin, S100A10 protein, two actin isoforms and Rap-1b were down-regulated. The only protein similarly expressed by both types of differentially matured DCs was GAPDH.

### 3.5 Validation of protein expression by flow cytometry

**Fig. 5:**
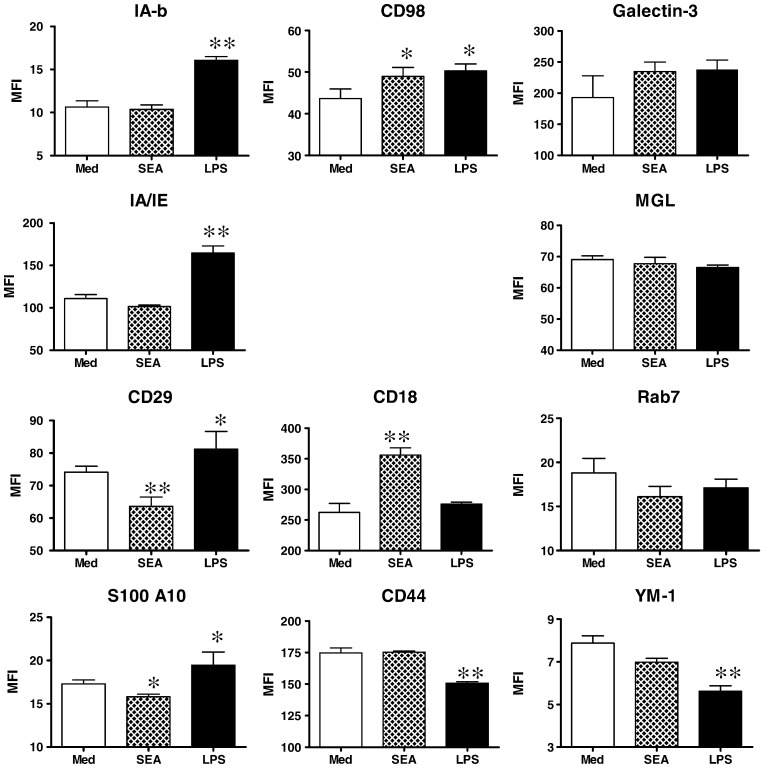
Validation of protein expression on DCs judged by flow cytometry of antibody labelled cells. Various molecules expressed by DCs were quantified by flow cytometry of antibody labelled cells. Bars are means of MFI values of three separate cultures for each type of differentially matured DC. Significant differences of SEA-DCs and LPS-DCs compared to MED-DCs as **p < 0.01 and *p < 0.05.

Several proteins identified by our iTRAQ studies, for which commercially available antibodies are available, were validated for changes in expression by flow cytometry (Fig. 5). The MHC class II molecules IA-b and IA/IE were both significantly up-regulated on LPS-DCs compared to SEA-DCs and MED-DCs, confirming the changes in expression recorded using iTRAQ. CD29 and S100A10 were also up-regulated on LPS-DCs but down-regulated in SEA-DCs, mirroring proteomic analysis. Whilst CD98 was up-regulated in both LPS-DCs and SEA-DCs compared to MED-DCs, CD18 was only up-regulated in SEA-DCs and was more abundant than in LPS-DCs. CD44 was expressed at higher levels in SEA-DCs than LPS-DCs. Several molecules were expressed at similar levels in all three types of DCs (*i.e.* galectin-3 and CD301b) corroborating proteomic analysis, although the expression of Rab-7 which was elevated at the proteomic level in SEA-DCs compared to LPS-DCs, was not differentially expressed as judged by flow cytometry. Finally, the secretory molecule YM-1, which was significantly down-regulated in LPS-DC *versus* MED-DC as determined by iTRAQ, was also down-regulated as judged by flow cytometry, although it was detected at slightly lower levels in SEA-DCs compared to MED-DCs.

## 4 Discussion

Over the past decade, proteomics has been utilised to determine differences between various immune cells [23] and in particular to study differences between whole cell extracts from immature DCs and differentially matured DCs with various PAMPs [24]. Mainly, these studies have employed 2-DE-based proteomics and highlighted changes in the expression of cytoskeletal and cytoplasmic molecules needed for basic cellular functions [12–14,25]. Such an approach favours soluble and abundant cytosolic proteins but compromises the detection of scarcer and detergent-soluble proteins, such as those present in plasma membranes. In the present study, we focussed upon the proteomes of plasma membrane-enriched fractions of differentially matured DCs. Although the fractions isolated were relatively crude, they were clearly enriched in plasma membrane components (~ 30–50%).

Indeed, LC–MS/MS has several advantages in the analysis of plasma membrane components. First, it requires only low amounts of any given protein for MS analyses, so is suitable for scarcer proteins. Second, digestion of enriched membranes can be performed in the presence of trace amounts of ionic detergents which, along with the use of reducing and alkylating reagents, allows for improved trypsin cleavage leading to higher sequence coverage for a given protein. Furthermore, labelling peptides with iTRAQ tags prior to LC–MS/MS allows the relative amounts of a protein to be determined in different cell groups, which is difficult to be accurately demonstrated by ‘label-free’ LC–MS/MS.

As revealed by our ‘label-free’ shotgun proteomics and found in all three types of DCs, the most abundant molecules associated with the plasma membrane including those with a CD prefix, ras-related proteins, MHC molecules and GNBPs. Others included proteins associated with membrane and vesicle trafficking such as the calcium-sensitive annexins [26] and phagocyte-specific S100 calcium binding proteins which are major damage associated molecular patterns [27]. The three differentially matured DCs also all expressed lymphocyte cytosolic protein 1 (also called 65 kDa macrophage protein), LSP-1 (F-actin binding protein) and FcεRI, although various immune associated proteins were only found in one or two of the DC types. For instance, immature MED-DCs and pro-Th2 SEA-DCs both expressed CD11b, CD44, CD45 and CD48, while both pro-Th2 DCs and pro-Th1 DCs expressed the heavy chain of CD98 which is involved in activation of naive and memory CD4 and CD8 T cells [28], thereby arguing against the ‘default’ hypothesis of pro-Th2 DCs.

The association of LC–MS/MS with iTRAQ labelling enabled comparison and quantification of changes in the plasma membrane proteome, many of which being confirmed by flow cytometry. As our study compared three biological replicates of each type of DCs, we were able to determine whether changes in the level of protein expression were reproducible. Our study revealed significantly increased levels of MHC class II molecules in pro-Th1 DCs, which were unchanged or decreased in pro-Th2 DCs. Surprisingly, CD29 which mediates the interaction of immature DCs with extracellular matrix components, and thus with a likely role in the retention of DCs in the periphery [29,30], was also highly expressed by LPS-DCs *versus* immature DCs. Conversely, pro-Th2 SEA-DCs expressed increased levels of proteins like CD18 and CD44. The greatest decrease of any protein expressed in pro-Th2 SEA-DCs was for NAP-22 (also called BASP-1 or CAP-23) whereas it was greatly up-regulated on pro-Th1 DCs. This molecule was originally identified as a membrane/cytoskeletal protein from rat brain and is a member of a family of motility associated proteins linked to actin reorganisation and neurite development [31,32]. Recently, it has been defined in the context of blocking oncogenic Myc protein-induced cell transformation [33]. Although this molecule was not expected to be expressed by DCs, and has not previously been identified as having demonstrable immune function, its differential distribution between pro-Th1 *versus* pro-Th2 is intriguing and warrants further investigation. One of the most down-regulated molecules in pro-Th1 LPS-DCs *versus* MED-DCs was YM-1 as determined from the proteomic data and verified by flow cytometry. This molecule has been associated with the development of ‘alternatively-activated’ macrophages and the recruitment of eosinophils [34], particularly after multiple exposure to schistosome cercariae [35] and other helminth infections which induce Th2-type immune responses [36,37].

A number of other changes in protein expression may help explain the differential DC phenotype/function. For example, SEA-DCs may be more efficient at antigen uptake and processing than immature MED-DCs as several chaperone proteins and enzymes involved in protein folding were up-regulated in pro-Th2 DCs compared to immature DCs (*i.e.* HSP-90α, two isoforms of hsc71, clathrin and Rab-7) although flow cytometry analysis revealed intracellular Rab-7 expression was similar between pro-Th2 DCs and pro-Th1 DCs. Another molecule up-regulated in SEA-DCs was protein disulfide isomerase which not only participates in the editing of MHC class I peptide repertoire [38] but also inhibits the transcriptional activity of NF-κB as a downstream signal of IL-10 [39]. In contrast, most of the proteins reported above were not identified in mature pro-Th1 DCs that have lost their capacity to take up and process antigen, however they are poised to present antigens, as suggested by our GO term enrichment analysis. Moreover, many enzymes and transporters related to general cell metabolism (*i.e.* malate dehydrogenase, pyruvate kinase 3, arginosuccinate synthase, fructose-bisphosphate aldolase A, enolase 1 and two chains of transporting ATPase Na^+^/K^+^) were up-regulated, or expressed at similar levels, between SEA-DCs versus MED-DCs, while their expression was mostly decreased in LPS-DCs versus MED-DCs. This suggests that MED-DCs and pro-Th2 DCs are more metabolically active compared to pro-Th1 LPS-DCs that have matured into an ‘end state’. GO term enrichment analysis confirmed that pro-Th2 DCs up-regulated proteins associated with nucleotide metabolism, whilst at the same time had down-regulated responses to chemical stimulus and regulation of the adaptive immune response.

Many of the proteomic changes in pro-Th2 SEA-DCs affected cytoskeletal proteins confirming previous studies using 2-DE-based proteomics [14]. For example, vimentin, tubulin β5, non-muscle myosin, talin and coactosin-like protein were all up-regulated in SEA-DCs, whereas proteins associated to actin such as Arp2/3 with a role in membrane trafficking [40] and ezrin–radixin–moesin complex that links cytoskeletal components with plasma membrane proteins [41] were down-regulated *versus* MED-DCs. Moreover, in LPS-DCs few cytoskeletal proteins were identified, alternatively they were down-regulated or expressed at similar levels compared to MED-DCs (*i.e.* actin isoforms, destrin and moesin). Finally, our study also reveals that SEA-DCs contain twice as many nuclear proteins than LPS-DCs or immature DCs. These proteins include initiation and elongation factors, several histones, signalling proteins (MAPK3, Erk-1) indicating that SEA may be a potent stimulus for DC proliferation.

Our study using iTRAQ labelling combined with LC–MS–MS has provided a valuable appraisal of the repertoire of proteins expressed by differentially matured DCs particularly within the plasma membrane. This offered new insights into how differentially matured DCs may function to promote Th1 or Th2 polarisation of the adaptive immune response. A caveat to studies using bone-marrow derived DCs is that whilst the cells have a high level of purity and are of immature/neutral status, they may not be representative of DCs from tissues *in vivo* which are subject to many local factors, such as the local cytokine environment [35], which influence their maturation as pro-Th1 *versus* pro-Th2 DCs. However, we believe that our combined approach has the potential to reveal the identity of further molecules involved in DC differentiation, especially if increased peptide sample fractionation coupled to higher sensitivity mass spectrometry instrumentation is employed. Accordingly, Segura et al. [42] recently reported an extensive comparative list of membrane proteins from immunopurified DCs using 2D liquid peptide fractionation followed by MS/MS in an Orbitrap instrument. However, their analysis was performed using only a single biological sample and protein expression was inferred by spectral counting. Their study also did not examine differential maturation after stimulation of DCs with different PAMPs.

In conclusion, our study and resulting analysis of the proteomic data support the view of a ‘limited maturation’ of pro-Th2 DCs compared to pro-Th1 DCs. We also report evidence of the expression of a restricted number of membrane proteins which may be unexpected ‘signatures’ of pro-Th2 DCs and consequently warrant further investigation.

The following are the supplementary materials related to this article.

Supp. Tables 1, 2, and 3 List of proteins identified by LC–MS/MS and their classification. Alphabetical list of proteins identified by LC–MS/MS in plasma membrane enriched fractions from (1) MED-DCs, (2) SEA-DCs, and (3) LPS-DCs. The accession numbers were given by the Mascot search against the CDS mouse database. Plasma membrane identities were in bold and shaded in grey. Italics indicate single-peptide proteins.

Supp. Table 4 Total protein hits identified by iTRAQ between SEA-DCs *versus* MED-DCs. Alphabetical order of the total hits derived from the six MS/MS peak lists from the three iTRAQ experiments comparing SEA-DCs to MED-DCs. For each protein identified, we indicate the number of replicates where it was identified (count); the accession number given by the Mascot search against the CDS mouse database; the number of peptides enabling protein identification; the total number of peptides; the index; the log (*ratio*) between MED1-DCs (C1) and MED2-DCs (C2); the log(*ratio*) between SEA-DCs (T) and MED1-DCs; the log(*ratio*) between SEA-DCs and MED2-DCs; the three respective *ratio* in the same order; the average log(*ratio*) between SEA-DCs and MED-DCs; the protein identity and its classification.

Supp. Table 5 Total protein hits identified by iTRAQ between LPS-DCs *versus* MED-DCs. Alphabetical order of the total hits derived from the six MS/MS peak lists from the three iTRAQ experiments comparing LPS-DCs to MED-DCs. For each protein identified, we indicate the number of replicates where it was identified (count); the accession number given by the Mascot search against the CDS mouse database; the number of peptides enabling protein identification; the total number of peptides; the index; the log*(ratio)* between MED1-DCs (C1) and MED2-DCs (C2); the log*(ratio)* between LPS-DCs (T) and MED1-DCs; the log*(ratio)* between LPS-DCs and MED2-DCs; the three respective *ratio* in the same order; the average log*(ratio)* between LPS-DCs and MED-DCs; the protein identity and its classification.

Supp. Table 6 Protein changes between pro-Th2 SEA-DCs *versus* immature MED-DCs revealed by iTRAQ labelling. List of proteins confidently identified by iTRAQ as up-regulated (average log(*ratio*)>0.046), unchanged (− 0.041<average log(*ratio*)<0.046), and down-regulated (− 0.041<average log(*ratio*)) between SEA-DCs and MED-DCs in order of most up-regulated at the top to the most down-regulated at the bottom. Rows shaded in grey highlight changes at the most stringent threshold: log*(ratio)* values of ± 0.1. The accession numbers were given by the Mascot search against the CDS mouse database. The average value is the mean between the three different log(*ratio*) values from three iTRAQ replicates. N is the number of MALDI plates where the protein was identified (2 for one replicate; 4 for two replicates and 6 for three replicates).

Supp. Table 7 Table 2. Protein changes between pro-Th1 LPS-DCs *versus* immature MED-DCs revealed by iTRAQ labelling. List of proteins identified confidently by iTRAQ as up-regulated (average log(*ratio*)>0.046), unchanged (− 0.041<average log(*ratio*)< 0.046), and down-regulated (− 0.041<average log(*ratio*)) between LPS-DCs and MED-DCs in order of most up-regulated at the top to the most down-regulated at the bottom. Rows shaded in grey highlight changes at the most stringent threshold: log*(ratio)* values of ± 0.1. The accession numbers were given by the Mascot search against the CDS mouse database. The average value is the mean between the three different log(*ratio*) values from three iTRAQ replicates. N is the number of MALDI plates where the protein was identified (2 for one replicate; 4 for two replicates and 6 for three replicates).

## Figures and Tables

**Table 1:** Experimental design for iTRAQ. Summary of the six experiments comparing plasma membrane proteins from separate cultures of DCs grown in medium only (MED #1–6), or stimulated with SEA (SEA #1–3) or LPS (LPS # 1–3).

iTRAQ Label			
Expt #	114	115	116
1	MED-1	SEA-1	MED-1
2	MED-2	SEA-2	MED-2
3	MED-3	SEA-3	MED-3
4	MED-4	LPS-1	MED-4
5	MED-5	LPS-2	MED-5
6	MED-6	LPS-3	MED-6

**Table 2:** Analysis of differentially regulated proteins according to Gene Ontology term. Proteins were analysed for GO term enrichment and presented as up-regulated (top panel), or down-regulated (bottom panel) for SEA-DCs and LPS-DCs. Significance is given as p-value, whilst the number of different proteins identified within a given classification is presented as k.

SEA-DCs *versus* MED-DCs				LPS-DCs *versus* MED-DCs			
Up-regulated				Up-regulated			
GO term		p-value	k	GO term		p-value	k
Cell projection	GO:0042995	1.49E−11	13	Antigen processing and presentation of exogenous peptide antigen	GO:0002478	1.27E−08	3
Purine ribonucleoside triphosphate binding	GO:0035639	1.34E−06	10	MHC protein complex	GO:0042611	1.67E−08	3
Metabolic process	GO:0008152	5.52E−05	17	Peptide antigen binding	GO:0042605	5.42E−06	2
Nucleoside phosphate metabolic process	GO:0006753	6.53E−05	5	Cellular developmental process	GO:0048869	9.77E−04	4

Down-regulated				Down-regulated			

GO term		p-value	k	GO term		p-value	k

Response to chemical stimulus	GO:0042221	8.38E−07	10	Active transmembrane transporter activity	GO:0022804	6.49E−05	3
GTPase activity	GO:0003924	2.02E−07	5	Catabolic process	GO:0009056	2.92E−04	4
Antigen processing and presentation of exogenous peptide antigen *via* MHC class II	GO:0019886	5.49E−05	2	Response to wounding	GO:0009611:	2.12E−04	3
Regulation of adaptive immune response	GO:0002819	6.44E−05	3				

**Table 3:** Extrapolated proteins changes between SEA-DCs and LPS-DCs. List of proteins present both in Table 1 (SEA-DCs *versus* MED-DCs) and Table 2 (LPS-DCs *versus* MED-DCs) with their respective average log(*ratio*): *(1)* and *(2)*. The extrapolated average log(*ratio*) for pro-Th2 SEA-DCs compared to pro-Th1 LPS-DCs equals *(1)*–*(2)*. Shaded rows highlighted major changes at the most stringent threshold: log*(ratio)* values of ± 0.1. The table presents the proteins from the most up-regulated at the top to the most down-regulated at the bottom. The accession numbers were the ones given by the Mascot search against the CDS mouse database.

Accession no.	SEA *versus* MED	LPS *versus* MED	SEA *versus* LPS	Protein hits
*Up-regulation*				
/:spt|P26041|	− 0.079	− 0.334	0.255	Moesin (Membrane-organising extension spike protein)
/:trm|Q80X37|	− 0.078	− 0.285	0.207	CD44
/:pir|S04847|	− 0.021	− 0.129	0.108	CD18, Integrin β-2 precursor (LFA-1/CR3/P150,95 β-subunit)
/:dbj|BAB23738.1|	0.072	− 0.037	0.108	Ras-related protein Rab-7

*No change*				
/:spt|P16858|	0.017	− 0.023	0.040	Glyceraldehyde 3-phosphate dehydrogenase (GAPDH)

*Down-regulation*				
/:rf|NP_077777.1|	− 0.062	0.021	− 0.082	Ras-related protein Rap-1B
/:trm|Q9CXK3|	− 0.107	− 0.017	− 0.090	Actin, α, cardiac
/:rf|NP_031419.1|	− 0.112	− 0.014	− 0.098	Actin, β, cytoplasmic
/:spt|P08207|	− 0.155	− 0.048	− 0.107	S100 A10, calpactin I light chain
/:pdb|1BZ9_B|	− 0.040	0.067	− 0.107	β-2-microglobulin precursor
/:trm|Q7TMN7|	− 0.038	0.086	− 0.124	Annexin A4
/:spt|P10107|	− 0.098	0.031	− 0.128	Annexin A1, calpactin II
/:trm|Q9TP17|	− 0.107	0.159	− 0.266	H-2 class II histocompatibility antigen, A β chain precursor (I-A)
/:trm|Q91XV3|	− 0.208	0.078	− 0.286	22 kDa neuronal tissue-enriched acidic protein, NAP-22, BASP-1
/:trm|Q91VU2|	− 0.062	0.269	− 0.331	~ transgelin 2 (SM22β)
